# Properties of Inorganic Polymers Based on Ground Waste Concrete Containing CuO and ZnO Nanoparticles

**DOI:** 10.3390/polym13172871

**Published:** 2021-08-26

**Authors:** Aikaterini I. Vavouraki, Iosifina Gounaki, Danae Venieri

**Affiliations:** 1 School of Mineral Resources Engineering, University Campus, Technical University of Crete, GR-73100 Chania, Greece; 2 Department of Agriculture, School of Agricultural Science, Hellenic Mediterranean University, GR-71004 Heraklion, Greece; 3 School of Chemical and Environmental Engineering, University Campus, Technical University of Crete, GR-73100 Chania, Greece; iosifina.gounaki@enveng.tuc.gr (I.G.); danae.venieri@enveng.tuc.gr (D.V.)

**Keywords:** inorganic polymers, ground waste concrete, nanoparticles, copper oxide, zinc oxide, antibacterial activity

## Abstract

The effect of copper oxide and zinc oxide nanoparticles (NPs) on the mechanical and thermal properties of ground waste concrete inorganic polymers (GWC IPs) has been investigated. NPs are added to GWC IPs at loadings of 0.1, 0.5, 1, and 2% *w*/*w*. The phase composition and microstructure of NPs GWC IPs have also been examined using X-ray diffraction (XRD), Raman spectroscopy and scanning electron microscope (SEM/EDS) techniques. Results show that the mechanical properties of GWC IPs are improved (23 MPa) due to addition of NPs (1% ZnO). In particular, GWC IPs embedded with 0.5% CuO and 1% ZnO NPs exhibited relatively improved compressive strength. The addition of NPs decreases the macroporosity and increases the mesoporosity of IPs matrix and decreases relatively the ability of IPs matrix to water absorption. The antimicrobial activity of GWC IPs doped with 0.5 and 1% CuO NPs against *E. coli* was also determined.

## 1. Introduction

Due to rapid urbanization the increase in demand for engineering infrastructures, 20% of all greenhouse gas emissions is attributed to construction and also to maintenance of the built environment. The cement industry is responsible for c.a. 8% of global CO_2_ emissions [1]. Thus, it is of great environmental challenge in (partial) substituting cement as a raw material by using Portland cement clinker (PC) with supplementary cementitious materials or with non-clinker based cements such as recycled aggregates. Up to 20% substitution of virgin aggregates with concrete waste is not considered to lower the new concrete’s properties and can be used for structural applications [2]. The main obstacle for recycling aggregates from concrete waste in new concrete is the low cost of virgin materials and also, the processing costs of demolition wastes to secure high-quality material for recycling. The recent Circular Economy Package, launched by the European Commission introduces a new perspective on waste management policy-making through sustainability principles [3]. Policy measures encompass taxes on virgin materials, the encouragement of green public procurement, taxes for landfilling, and end-of-waste criteria, among others. Scrivener et al. [4] stated that increasing the average level of PC substitution in cement to 40% could avoid up to 400 Mt CO_2_ emissions globally each year.

Ground waste concrete (GWC) makes up part of the debris of construction and demolition wastes (CDW) that are generated during the construction, renovation, and demolition of buildings, roads, and other engineering works. About 374 million tons of CDW were generated in 2016 making CDW the largest waste stream in the EU by weight [5]. In Greece CDW account for approximately 4 million tons per year [6]. Construction and demolition is defined as a priority area in the EU according to the Circular Economy Action Plan [3]. Toward a circular economy the lifecycle of construction products may include preservation of resources and hence closing the loop. In other words, CDW management in the long term is of great importance providing significant environmental benefits such as waste prevention and/or less waste generated. Under the recycling concept, the partial substitution of cement with alternative raw materials such as CDW may lead to significant CO_2_ savings in the future.

Inorganic polymers (IPs) are considered as the third generation of concrete, since they are viable for the preparation of cements, mortars, and concrete with properties similar or higher than traditional materials used in constructions [7]. Furthermore, IPs have a lower carbon footprint compared to PC [8,9]. IPs have good and prospective properties over the PC system with regard to excellent mechanical properties, good chemical and fire resistance, low shrinkage, environmentally friendly nature, and long-term durability [10,11]. Previous studies focus on the valorization of CDW and/or industrial slags through inorganic polymerization [12,13,14,15,16]. Various application potential of IPs including toxic waste immobilization, building, and high temperature-resistant materials have been recently extensively reviewed [17,18].

One way to develop and modify IPs properties is the addition of nanoparticles (NPs). NPs possess unique physical and chemical properties due to high surface area and nanoscale size [19]. Good absorbability, mechanical properties, optical activity, and chemical reactivity with high dispersibility in alkaline solutions make NPs suitable materials for different applications. Singh et al. [20] demonstrated that the incorporation of different NPs (carbon nanotubes, nano-SiO_2_, -TiO_2_) in IPs mortars/concrete exhibited significant potential to be used as an effective building material in civil engineering field applications. In a recent review Rashad et al. [21] underlined the effect of NPs namely nano-SiO_2_, -TiO_2_ -Al_2_O_3_, -clay, and -silica waste into different IPs matrices. Specifically, NPs’ unique properties lead to their use as filler in IPs. There is an optimum concentration of NPs added in different-based IPs (i.e., fly ash-, metakaolin-, and slag-based IPs) that contribute to a better mechanical performance due to physical filler effect of NPs [22,23,24,25]. Thus, a relatively compact microstructure of IPs is achieved. The inclusion of NPs densifies the microstructure of IPs composites producing high mechanical strength. Due to the formation of a dense NPs-embedded-matrix of IPs gel interconnectivity of micropores is hindered [26]. There is an increase of scientific interest to synthesize high-quality IPs with better mechanical, structural, and thermal properties and NPs could be used for that purpose [27,28]. However, a relatively modest number of studies appear to elucidate the incorporation of CuO and ZnO NPs into IPs.

Among the most commercialized metal-based NPs, CuO and ZnO NPs (and/or nanofluids) are semiconductor materials suggested for various applications with technological perspectives (energy conversion and, storage, solar collectors) [29,30] to environmental remediation due to their antimicrobial activity, (photo)catalytic potential, removal of pollutants dyes, and heavy metal ion sensing [31,32]. For cement -based materials NPs accelerate the hydration of cement, decrease the porosity, and improve the pore structure of concrete [33,34,35]. CuO and ZnO NPs among others have already been proposed for improving physicochemical properties of cement [36,37] and also operating as antibacterial cement admixtures for the production of cement -based composites [38].

Recently, NP-embedded concrete and NP-incorporated coatings with novel functionalities such as self-protection and anti-corrosion ability have been successfully developed for prevention and control of biogenic deterioration of concrete [39]. As already mentioned IP mortars are currently considered as being more environmentally friendly materials and may be a favorable contender to Portland cement concrete. Hence, it is of great research interest to examine IPs composites with embedded -NPs as antimicrobial agents. The addition of nanosilver improved the mechanical strength, durability, and antibacterial properties of Ag-IPs composites [40,41,42]. Additionally, CuO and ZnO NPs can be included as reinforcement for IPs pastes [43,44] and also, generating antibacterial IPs surfaces [45,46].

Νanoparticles in inorganic polymer preparation is very limited. To the best of our knowledge this is the first attempt to use synthesized CuO and ZnO NPs embedded in ground waste concrete-based IPs. The aim of this study was to determine the effect of both synthetic CuO and ZnO NPs on physicochemical and thermal properties of GWC IPs. In particular the structural, mechanical, and thermal behavior of GWC IPs admixed with different concentrations of CuO and ZnO NPs (0.1 to 2%) was examined. The mechanical properties and microstructure of the produced IPs were determined through XRD, Raman, SEM/EDS, and TG/DTA analyses. The antibacterial potential of NPs GWC IPs was also investigated against Gram-negative *Escherichia coli* bacteria. Toward a better green construction technology GWC IPs binders are considered eco-friendly, non-hazard, cost effective, and more durable building materials that could be used in constructions, repair, and restoration cases [47,48]. Additionally, possible utilization of IPs embedded with NPs with antibacterial activity may be considered for functional surface applications such as floors and walls in houses and hospital facilities and/or marine concrete structures.

## 2. Materials and Methods

### 2.1. Synthesis of CuO and ZnO Nanoparticles

Dispersed CuO and ZnO NPs were obtained by co-precipitation of hydroxycarbonates from corresponding crystalline solids of Cu (NO_3_)_2_·3H_2_O (Sigma-Aldrich, Burlington, MA, USA) and/or Zn (NO_3_)_2_·6H_2_O (Alpha Aesar) and NaHCO_3_ (Alpha Aesar, Stoughton, MA, USA). The latter solution was added drop-wise in metal nitrate solutions under vigorous (magnetic bar) stirring rate (c.a. 300 rpm) at 65 °C for 3 h. Following filtration (membrane filters 0.45 μm cellulose acetate), the synthesized hydroxycarbonates powders were dried overnight at 80 °C (Jeio Tech, Des Plaines, IL, USA, ON-02G) and calcined in a pre-heated oven (N-8L SELECTA) at 350 °C (at a heating rate 10 °C·min^−1^) for 5 h to obtain the corresponding metal oxides. CuO and ZnO NPs were placed in distilled deionized water (10 mg·L^−1^) and subjected to sonification for 5 min to reduce agglomeration before characterization. NPs were characterized by XRD, BET, and SEM/EDS in order to determine phases, surface area, size, and morphology. Synthesis of CuO and ZnO NPs was previously reported by Carbone et al. [49].

### 2.2. Preparation of Inorganic Polymers

Ground waste concrete (GWC) was obtained from samples used for educational purposes at the Applied Mechanics Laboratory (School of Architecture, Technical University of Crete) and was pulverized in a Sepor type rod mill. GWC IPs were synthesized after alkaline activation of GWC with sodium hydroxide (NaOH 8 M) and sodium silicate solutions (Na_2_SiO_3_: 27% SiO_2_, 8% Na_2_O, mean values, Merck, Darmstadt, Germany). The ratio of liquid to solid was 0.3 and the ratio of NaOH to Na_2_SiO_3_ solution was 0.9. The produced pastes were cast for 3 h and, heated at 80 °C for 24 h, followed by curing for 7 days. Details of the experimental procedure and methodology of alkali-activated polymerization are described in a previous study [16]. At similar conditions GWC IPs embedded with CuO and/or ZnO nanoparticles were prepared at loadings of 0.1%, 0.5%, 1%, and 2% NPs.

### 2.3. Structure Analysis and Characterization

XRF (XRF-EDS, Bruker-AXS S2 Range type, and SPECTRO X-LabPro, USA) analysis was used to determine the composition of the GWC raw material. The uniaxial compressive strength of GWC IPs embedded with NPs was determined with the use of a MATEST C123N load frame. XRD, Raman, and SEM/EDS analyses are also performed focusing on the micro/nanostructure, morphology, and phase/surface elemental compositions of the produced IPs. Additionally BET, mercury intrusion porosimetry, and TGA were also carried out. The physical properties of the selected IPs were also determined according to Archimedes principle.

The D8-Advance Bruker instrument, using Cu-Kα1 radiation at 40 kV and 40 mA, was used for XRD analysis. The diffractograms were recorded at 20° to 80° (2θ range), with a 0.02° step size. The crystalline phases were identified using DIFFRACplus EVA^®^ software Bruker-AXS based on the ICDD Powder Diffraction File.

Raman spectra were recorded on a T-64000 (Jobin Yvon-Horiba, Glasgow, UK) micro-Raman system equipped with a 2D-CCD Symphony II detector. The excitation wavelength (514.5 nm) was provided by a DPSS laser (Cobolt Fandango TMISO laser). The laser power on the sample was maintained at 1.2 mW and was focused on the samples by a 50× microscope objective. The scattered beam passed through an appropriate edge filter (for the removal of the strong elastically scattered photons) and directed into the slit of the monochromator in the single spectrograph configuration. The Raman photons were dispersed by a 600-grooves/mm blazed holographic diffraction grating. The resolution was kept constant for all experiments (~7 cm^−1^). The spectrometer was wavenumber calibrated using the standard Raman band positions of Si reference sample. For each sample, a minimum of three spots were measured. The Raman spectra were recorded in the region of 160–2200 cm^−1^.

A field emission scanning electron microscope, SEM (FE-SEM, Leo Supra 35VP, Germany) equipped with an energy dispersive X-ray (EDX, QUANTA 200, Bruker AXS, Billerica, MA, USA) with an accelerating voltage of 20 kV was used for morphological examination. In order to improve image quality and obtain high resolution images, the samples selected for SEM analysis, were coated with gold using a sputter coater.

The physical properties of GWC IPs embedded with NPs were determined according to the Archimedes principle as described in a previous study [16]. Thus, the apparent porosity and relative density and also, water absorption of samples were determined.

The Brunauer-Emmet-Teller (BET) surface area and pore analysis were carried out with a NOVA Surface Area Analyzer (Quantachrome instruments, Boynton Beach, FL, USA) using nitrogen as the adsorbent gas at 77 K. Prior to these measurements, GWC IPs embedded with NPs samples (about 1 g) were degassed at 473 K overnight under the vacuum. Gas adsorption analysis in the relative pressure range of 0.05 to 0.3 was used to determine the total specific area. Surface areas were calculated with an accuracy of 10%, from the isotherm data using the BET method. The total pore volume of the samples was calculated using t-plot analysis. The Barrett-Joyner-Halenda (BJH) method was used to obtain the average pore size.

Macroporosity of IPs was examined by mercury intrusion porosimetry. Open porosity and bulk density of selected samples were determined by Micromeritics Autopore IV 9400 porosimeter (Atlanta, GA, USA).

TG/DTG analyzer (Model TGA 6 Perkin Elmer, Waltham, MA, USA) was used to carry out differential thermal analysis. The thermogravimetric analysis was performed up to a maximum temperature of 900 °C, at a heating rate of 10 °C·min^−1^ and a nitrogen atmosphere with a 80 mL·min^−1^ flow rate.

### 2.4. Antibacterial Activity

The antibacterial properties of GWC IPs with varied amounts of CuO and ZnO-NPs (0.5, 1, and 2%) were tested against a reference strain of *Escherichia coli* (DSM 498). Samples were prepared in the form of disks (Ø 20 mm) and they were designated as Cu0.5, Cu1, Zn1, Zn2 for 0.5%, 1%, and 2% doped GWC IPs. Due to high alkalinity of alkali-activated samples (c.a. pH 12) disks of GWC IPs with NPs were immersed in HCl 0.1 M twice (with fresh solution) for 1 h and used further for antibacterial study purposes.

Tests of bacterial inactivation were performed in nutrient broth (LABM) with a bacterial concentration of 10^5^ CFU/mL. Specifically, samples were added in 6 mL of liquid culture, which was further diluted with NaCl (0.7% *w*/*v*) to a final volume of 50 mL, in order to obtain the desired bacterial concentration (105 CFU/mL). All mixtures were incubated at 37 °C for 24 h. After the incubation period, the optical density of each liquid culture was measured at 600 nm and viable counts were performed applying the spread plate technique and using nutrient agar (LABM). Total of 200 μL of each liquid culture was spread onto nutrient agar plates, followed by incubation at 37 °C for 24 h. Bacterial reduction was evaluated through colony counting. Moreover, uncoated beads were tested as control samples in order to investigate any effect on bacterial growth. The antimicrobial potential of NPs GWC IPs (in disks) was expressed as *E. coli* growth per sample weight and area (*E. coli*/kg sample/m^2^). Finally, metal ion concentration (namely copper or zinc) in the same liquid culture that was used for bacterial growth, was determined with the use of inductively coupled plasma mass spectrometry (ICP-MS) (Agilent Technologies, Santa Clara, CA, USA, 7500cx).

## 3. Results and Discussion

### 3.1. Characterization of Nanoparticles

**Figure 1:**
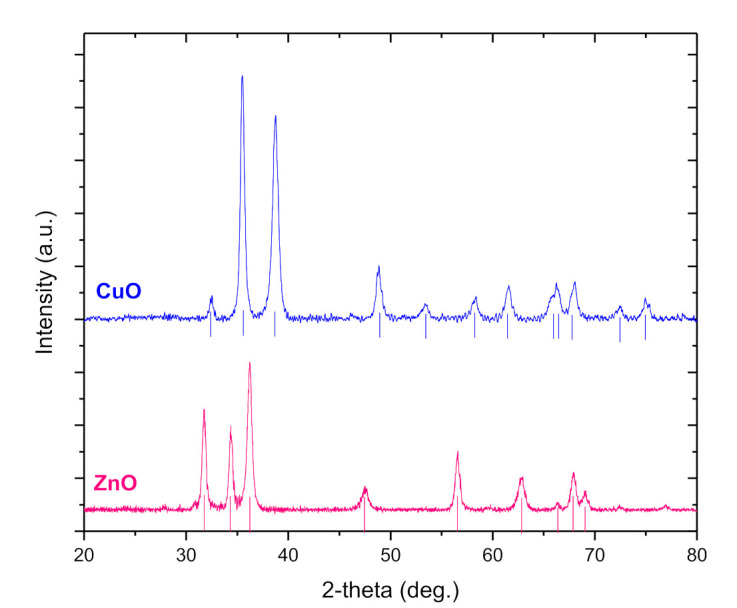
XRD patterns of CuO and ZnO NPs; straight lines indicate the match to the reference patterns of CuO and ZnO.

Figure 1 shows the powder XRD patterns of CuO and ZnO NPs and the observed peaks were in excellent agreement with the standards (JCPDS Pdf No. 44–706 and 36–1451 for tenorite, CuO and zincite, ZnO, respectively). The particles were well crystallized.

The average nanocrystallite size of NPs was estimated by using Deybe-Scherrer equation [50]:

(1) $$D=K\frac{\lambda }{B\cos\theta }$$

where, *λ* is the X-ray wavelength (0.154 nm), *K* is Scherrer constant (0.9), and *B* is the full width at half maximum intensity (FWHM in rad); *θ* is the Bragg angle (in rad).

The size of the obtained CuO and ZnO NPs is calculated 20 and 16 nm, respectively. BET analysis determined the surface area of CuO and ZnO NPs that were 24.6 and 37.8 m^2^∙g^−1^, respectively in agreement with previous study [49].

**Figure 2:**
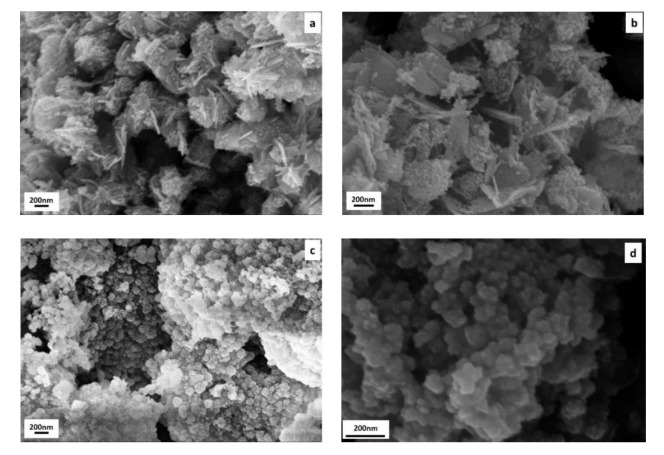
SEM images of (**a**,**b**) CuO and (**c**,**d**) ZnO NPs.

The morphology and the size of the produced CuO and ZnO nanoparticles were investigated by SEM/EDS (Figure 2). NPs were organized into spherical assemblies as (agglomerated) NPs aggregates. CuO NPs formed platy cluster (like dandelion) (Figure 2a,b) while ZnO NPs have the shape of faceted crystals (Figure 2c,d), as previously reported [49,51,52]. Both synthesized CuO and ZnO were less than 200 and 100 nm, respectively in size. The size of NPs was higher in comparison to the estimated average size of NPs obtained by XRD. Despite sonication, the NPs were agglomerated as seen in SEM images and thus, difficult to obtain accurate size measurements. Additionally high size of particles may be due to the fact that SEM images were taken for very small portion of the sample. Differences in average size obtained by XRD and SEM analysis have been previously reported [53].

### 3.2. Characterization of Inorganic Polymers from Ground Waste Concrete with Nanoparticles

Table 1 presents the elemental oxide composition analysis of raw material of ground-waste concrete (GWC) by oxides, according to XRF analysis. GWC is a Ca-rich material with low amounts of SiO_2_ (3.96%) and Al_2_O_3_ (0.99%).

#### 3.2.1. Mechanical Strength

**Figure 3:**
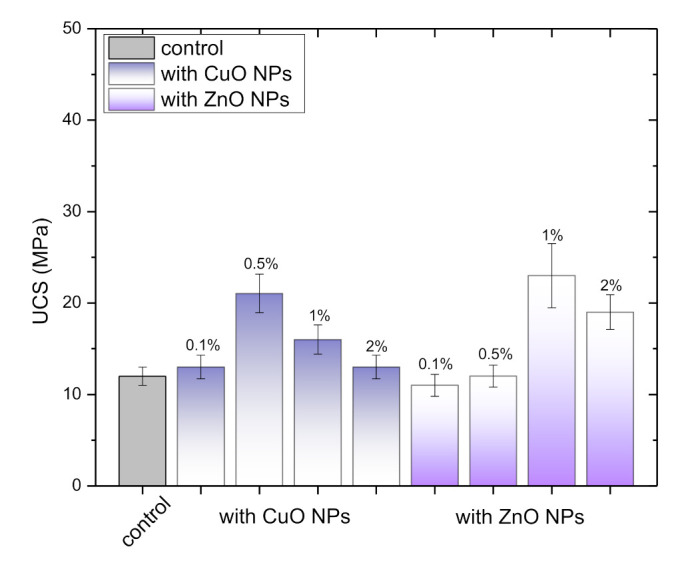
Compressive strength of CuO and ZnO NPs blended GWC IPs specimens (control: GWC IPs). Replicates of two IPs specimens (~10% standard error).

The compressive strength of GWC IPs embedded with varied amounts of CuO and ZnO NPs (0.1 to 2%) was measured (Figure 3). GWC IPs specimens have low compressive strength. Despite the fact that the SiO_2_ to Al_2_O_3_ molar ratio is high in the initial paste (6.8) both SiO_2_ to CaO and SiO_2_ to (Al_2_O_3_ + CaO) molar ratios are quite low (0.07). Thus, limited alkali activation is expected with few dissolved Si and Al ions for aluminosilicate bonding (Vavouraki, 2020). GWC IPs with 1% ZnO NPs had relatively higher compressive strength (23 MPa) compared to GWC IPs with and without CuO NPs. When the amount of ZnO added in GWC IPs increased (2%) the compressive strength decreased.

Previous studies investigated the effect of different NPs on the mechanical properties of IPs. The incorporation of 0.5% ZnO NPs and 2% Al_2_O_3_ NPs (of particle size of 60 nm and 300–500 nm) increased compressive strength of metakaolin-based IPs by 26% and 30%, respectively [44,54]. Metakaolin geopolymer CuO-NPs composites gained improved flexural strength [43]. Silver (4 nm)-silica (20–40 nm) nano-composite-modified fly-ash-based geopolymer mortars had higher compressive strength compared to conventional (OPC) cement mortar [40]. The inclusion of nano-silica from 0 to 3% in the OPC and GGBFS blended IPs mortars increased compressive strength by 40 to 64% as compared to the corresponding control mixes [22].

#### 3.2.2. XRD Analysis

**Figure 4:**
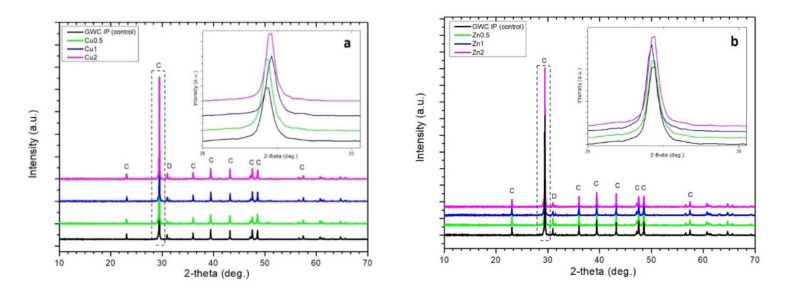
Powder X-ray diffraction patterns of GWC IPs (control) embedded with different amounts (0.5, 1%, and to 2%) of (**a**) CuO and (**b**) ZnO NPs; C: calcite; D: dolomite depict the match to the reference pattern of calcite, CaCO_3_.

Figure 4 shows the XRD patterns of CuO/ZnO NPs and GWC IPs (control) with different amounts of CuO/ZnO NPs added. The presence of the main crystalline phases calcite and dolomite in GWC was identified (JCPDS Pdf No. 5–586 and 36–426 for calcite, CaCO_3_ and dolomite, CaMg(CO_3_)_2_, respectively). The spectra exhibit intense peaks at 29.4° (2θ) corresponding to calcite. Limited amorphicity is indicated as no obvious hump is shown in the XRD patterns; thus, limited aluminosilicate gel is formed during geopolymerization justifying the low strength of GWC IPs. A small shift to higher angles is observed when adding NPs. This can probably be attributed to an interaction with the freely dissolved species [44].

#### 3.2.3. Raman Analysis

**Figure 5:**
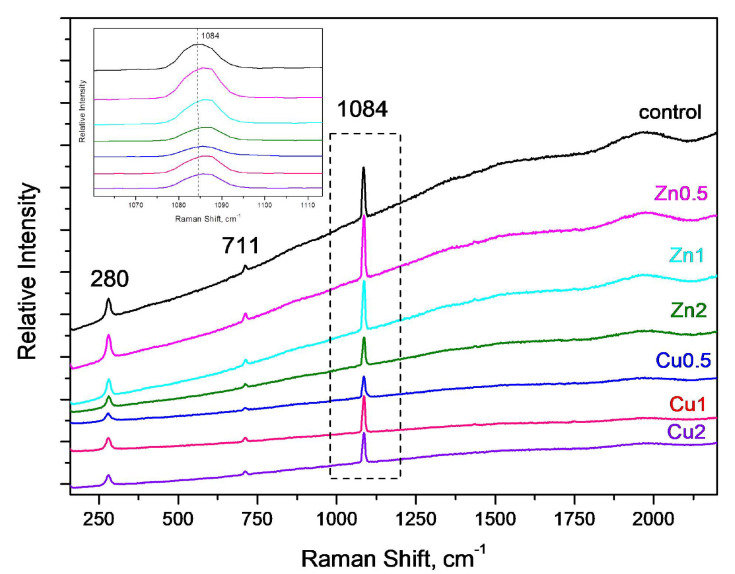
Raman spectra of GWC IP (control) embedded with different amounts (0.5 to 2%) of CuO and ZnO NPs; focused area at 1084 cm^−1^.

Figure 5 shows the Raman spectra of GWC IPs embedded with CuO and ZnO NPs. The characteristic vibrational signature of the calcite-type spectrum is present corresponding to symmetric stretching of CO_3_ group at 1084 cm^−1^ and ν4 symmetric bending mode at 711 m^−1^, and also the band at 280 cm^−1^ attributed to external vibrational modes of the CO_3_ groups that involve relative translations between the cation and anionic group [55,56]. Due to poor aluminosilicate content of raw material, GWC low strength of GWC IPs is anticipated, thus inorganic polymerization is limited [16]. Vibrational frequencies of silicate species for Raman spectroscopy, typically found in aluminosilicate-matrix-IPs have been previously reported [57]. The 1050–1100 cm^−1^ region may also be assigned to the asymmetric stretching vibrations of Si-O-Si or Al-O-Si. In this study the main band was centered at 1084 cm^−1^ that may hinder limited vibrations of Si-O-Si and Si-O-Al linkages. The broadening of the 1084 cm^−1^ band and its shift to a slightly higher wavenumber may suggest formation of a differently structured material that is associated with limited geopolymerization and/or NPs addition into IPs matrix [56]. A small shift of the intense peak may confirm the incorporation of nano-ZnO into the geopolymer matrix. It is worth noting that there is neither evidence of CuO or ZnO Raman signatures nor new functional group after the incorporation of CuO/ZnO NPs, indicating that only a physical interaction occurred between NPs and IPs matrix [44].

#### 3.2.4. SEM/EDS Analysis

**Figure 6:**
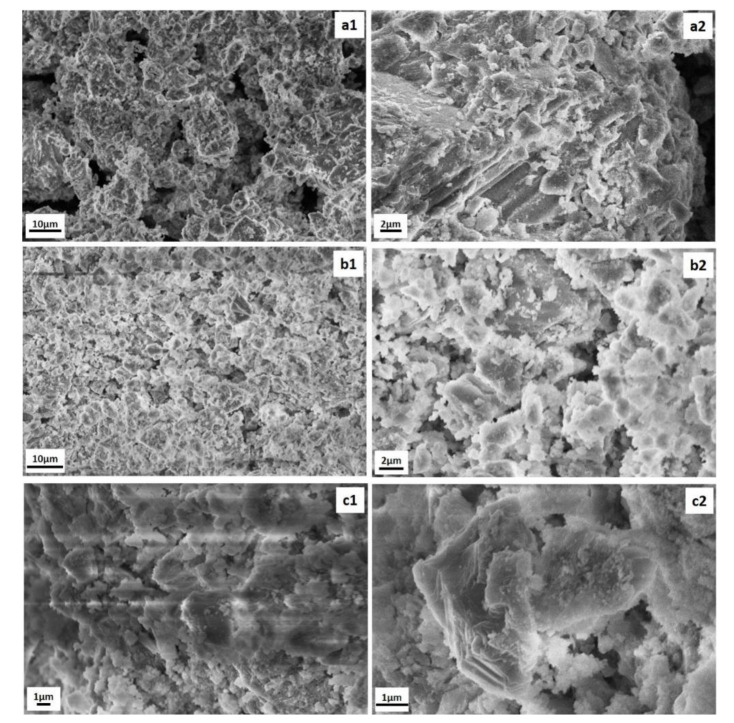

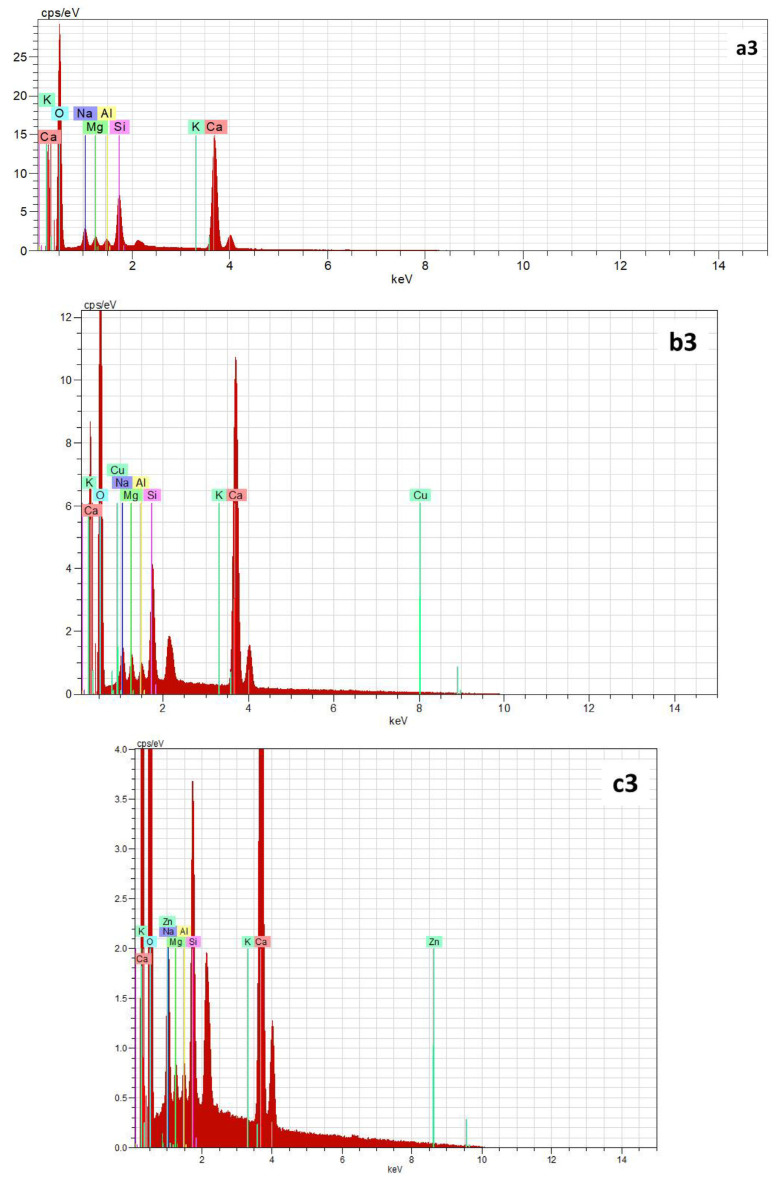
SEM images of (**a1**,**a2**) GWC IPs (control); (**b1**,**b2**) with 0.5% CuO NPs and, (**c1**,**c2**) with 1% ZnO NPs; and (**a3**,**b3**,**c3**) EDS analysis.

Figure 6 shows the scanning electron microscopy (SEM) images of GWC IPs embedded with 0.5% CuO NPs and 2% ZnO NPs. GWC IPs (control) have a porous structure with non-reacted particles (Figure 6(a1)). Along the step edges of calcite (as identified in XRD analysis) there is limited gel formation (Figure 6(a2)). Incorporation of CuO (Figure 6(b1,b2)) and ZnO NPs (Figure 6(c1,c2)) improved the homogeneity of GWC IPs matrix, forming a denser structure. This may be attributed to the filling of the matrix voids. Similar observations have been reported in previous studies [22,44]. NPs may act as fillers in alkali-activated materials improving particle packing with reduced porosity and increased compressive strength.

EDS was also performed to identify the chemical constituents present in GWC IPs. The elemental composition of GWC IPs embedded with 0.5% CuO and 2% ZnO NPs are shown (Figure 6(a3,b3,c3)). EDS measurements (Table 2) reveal the presence of O, Ca, Si, Na, Mg, Al, K, and relatively low amounts of Cu and/or Zn. Gold was also detected as a result of the gold coating process. Similar compositional analysis within samples indicate that the formation of the limited gel was not hampered by the addition of NPs and the latter coexist in the structure of the GWC IP matrix. Similar observations were obtained following the addition of titanium oxide particles into metakaolin-based IPs [23]. The presence of copper and zinc in GWC IPs is attributed to the addition of NPs into GWC IP matrix during the preparation of the samples. Copper content in GWC IPs (0.66% Cu or 0.41% CuO) is in good correlation with the initial content (0.5% CuO) added during the geopolymerization process. However, this was not that the case for zinc (just 0.03% Zn or 0.04% ZnO compared to 2% ZnO initially). This may be due to the fact that the main sodium and zinc peaks overlap in EDS spectra.

#### 3.2.5. Pores, Water Absorption, Sorptivity

**Figure 7:**
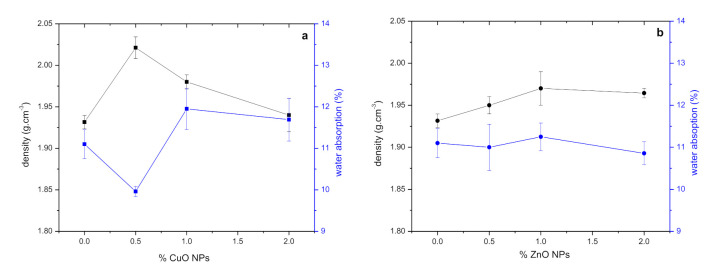
Density and water absorption of GWC IPs with different amounts of (**a**) CuO and (**b**) ZnO NPs.

Figure 7 shows the density and water absorption of GWC IPs with different amounts of NPs. GWC IPs with 0.5% CuO NPs and 1% ZnO NPs exhibit a relative increase in density and reduction in water absorption. The porosity results are in agreement with the compressive strength results following the addition of NPs. Further addition of NPs in GWC IPs resulted in a decrease of density and increase of water absorption.

Table 3 shows the physical characteristics of GWC IPs, namely specific surface area; total pore volume and average pore diameter in the presence of CuO and ZnO NPs. The average pore size obtained for GWC IPs is 87 Å; thus, it is classified as predominantly mesoporous material (mesoporosity range 45 to 500 Å). The medium pore volume and surface area for the GWC IPs indicate that IPs matrix is relatively dense with relative low permeability and relative high durability [58]. NPs incorporation into GWC IPs resulted in marginal difference in specific surface area and pore volume analysis. However, the average pore diameter (mesoporosity) of GWC IPs matrix embedded with NPs was increased by 42% (from 87.5 to 125 Å, mean value). This is an important result that correlates with compressive strength data (Figure 3). The addition of NPs into GWC IPs matrix resulted in the augmentation of the mesoporosity of matrix and thus an increase of compressive strength of the matrix was expected. In general, mesopores represent the voids between the IPs phases, while micropores primarily are within the gel network [59]. During alkali-activation process, the gel fills the cracks/voids between unreacted GWC particles, aggregates the pore space in the IPs matrix, thus refining the size of the pores. In this study, mesoporosity of IPs matrix was increased by the addition of NPs.

#### 3.2.6. Hg-Porosimetry Analysis

**Figure 8:**
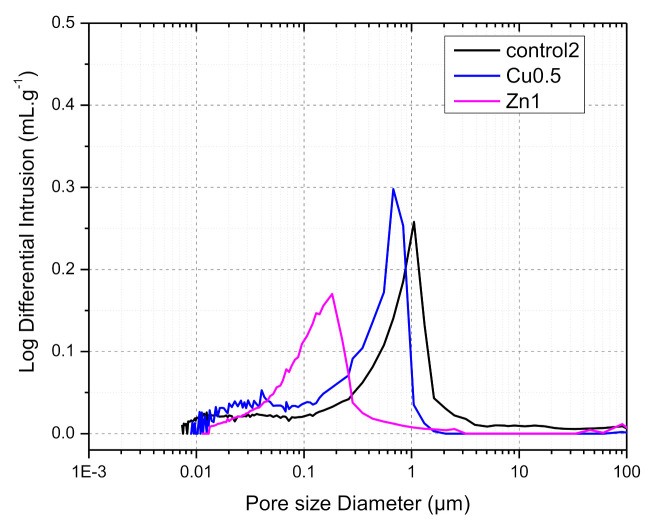
Pore size distribution (intrusion log differential, mL∙g^-1^ vs. pore size diameter, µm) of selected samples of GWC IPs (control) with 0.5% CuO and 1% ZnO NPs.

For selected samples of 0.5% CuO NPs and 1% ZnO NPs GWC IPs mercury intrusion porosimetry was performed; thus, pore size distribution curves were defined (Figure 8) and compared to GWC IPs (control; without NPs). The addition of NPs into IPs affected the porosity and pore distribution of IPs by increasing the number of small pores. The pore size distribution of GWC IPs (control) presented only one peak around 1 µm (range of capillary pores). The addition of 0.5% CuO and 1% ZnO NPs resulted in a shift to lower pore size (0.7 and 0.2 µm, respectively) compared to control, possibly due to the extent of filling the voids of GWC IPs. The mercury intrusion volume (area under the curve) was comparable for the control and 0.5% CuO NPs GWC IPs. However, this was not the case for 1% ZnO NPs GWC IPs; total intrusion volume was lower due to better void filling effect of ZnO NPs compared to CuO NPs. In agreement with compressive strength results (Figure 3), the lower the pore size of GWC IPs with 1% ZnO the higher the compressive strength. During SEM/EDS imaging (Figure 6b,c) poor development of IPs gel was observed, thus capillary pores are possibly formed by the space between unreacted GWC particles.

The average pore diameter of the selected samples was decreased from 0.125 µm to 0.111 µm and 0.093 µm (Table 4) as also shown by the porosity measurements of GWC IPs (from 26.5 to18.5 and 23.5%, for 0.5% CuO and 1% ZnO NPs GWC IPs, respectively). In general, the presence of macropores larger than 0.2 µm is characteristic of less reacted IPs which was the case in our study. The macropores fill the gaps between unreacted GWC particles [59]. Large capillary pores (>0.1 µm) initially found in the GWC IPs (control) became middle capillary pores (within 0.05–0.1 µm) for CuO NPs GWC IPs. NPs filled the pores of IPs, as already confirmed by SEM images.

The porosity decreased proportionally with increasing mechanical strength, in agreement with mechanical strength data. The reduction in the porosity by including an appropriate amount of NPs could be relevant in that NPs can refine pore structure by the filling effect and form more reaction products [21]. The incorporation of NPs improved microstructure of IPs possibly by chemical contribution of NPs through their high reactivity due to their high specific surface area values and/or the ability of unreacted NPs to fill IPs paste/interfacial transition zone (ITZ) voids [24]. Pore size is inversely proportional to the cross-linking density. Bulk density is defined as the total mass divided by the total volume (particles plus voids) [60], therefore a decrease in the pore volume means an increase in the bulk (or apparent) density. For 1% ZnO NPs GWC IPs high value of bulk (or apparent) density (2.09 and 2.74 g·mL^−1^ respectively) was obtained with a low value of void volume. IPs with high density generally result in high strength and a lower amount of voids and porosity. IPs with small voids show low (water and soluble elements) permeability [61]. Specific surface area (7 m∙g^−1^, mean value) obtained by BET method is in a good agreement with the area measured by Hg porosimetry (5.2 m∙g^−1^, mean value) [62].

#### 3.2.7. Thermogravimetric (TG) Analysis

**Figure 9:**
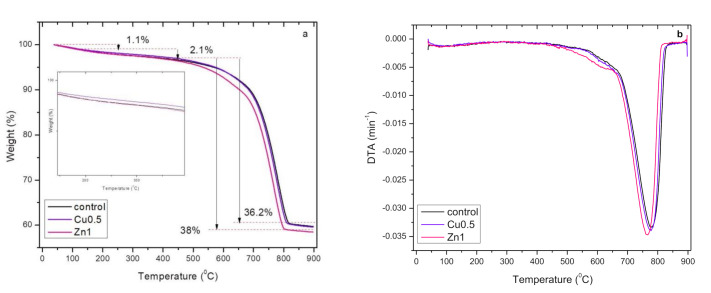
(**a**) Thermogravimetric (TG) analysis of GWC IP embedded with 0.5% CuO and 1% ZnO NPs; (**b**) DTA: derivative thermogravimetric analysis, the ratio of mass loss rate of sample to initial mass (min^−1^) measured.

The thermal properties of GWC IPs embedded with 0.5% CuO and 1% ZnO NPs was determined by TG analysis and the thermal decomposition of the materials was calculated. The spectra were divided into four different regions (Figure 9a). The first region up to 120 °C shows the weight loss of samples attributed to evaporation of physically adsorbed water; c.a. 1.1% of its initial mass. In the second region from 120 to 440 °C, weight loss of samples was c.a. 2.1% at a constant rate. However, water evaporation may still occur at higher temperatures due to the fact that remaining water is either bound tightly in pores or is less able to diffuse to the surface. The water weight loss is attributed to the de-hydroxylation of the chemically bound silicon-hydroxyl group giving (silicon-oxygen-silicon) bridge with loss of water [15,63]. Limited weight loss of hydrated sodium aluminosilicate gel justifies limited gel formation thus low compressive strength of GWC IPs. GWC IPs embedded with NPs showed lower thermal stability compared to the control. Despite low macroporosity of GWC IPs embedded with NPs, mesoporosity is high thus their ability to retain strong physically bonded water is reduced. The curve of 0.5% CuO NPs GWC IPs shifted slightly to a higher temperature (Figure 9a, zoomed area) than the control. This may be attributed to the effect of NPs filling the voids, producing relatively dense IP. In the third range (440 to 800 °C) chemically bound water begins to be removed [64], indicating the decomposition of calcium carbonate to calcium oxide [65]. The weight loss of 1% ZnO NPs GWC IPs was higher (38%) (Figure 9a) compared to GWC IPs (control) with 0.5% CuO (36.2%). The decrease in weight of ZnO NPs GWC IPs was at a relatively high rate. These changes can be attributed to the absorption of water residing in the channels of the matrix by NPs due to high surface area of NPs [44]. In the fourth region (800 to 900 °C) no further weight loss was observed. The curve of GWC IPs embedded with 1% ZnO NPs is steeper at lower temperature compared to the control, as presented in DTA graph (Figure 9b). These results are in agreement with BET analysis where the mesoporosity of IPs matrix was increased by the addition of NPs and thus the ability to retain water is diminished.

#### 3.2.8. Antibacterial Activity

**Figure 10:**
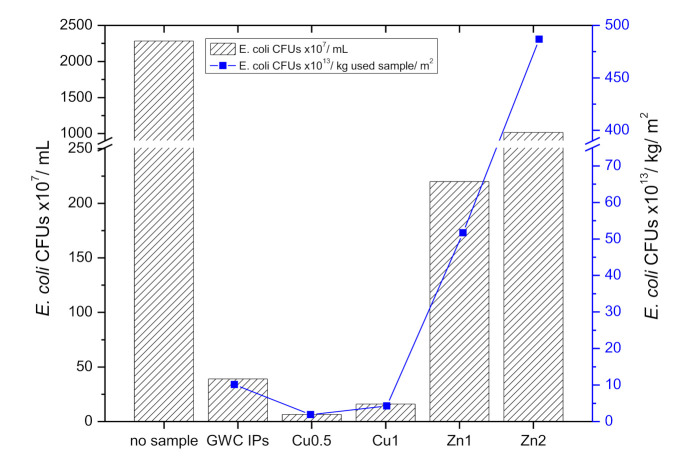
*E. coli* bacteria growth in the presence of GWC IPs embedded with CuO and ZnO NPs (*E. coli* CFUs/mL); (*E. coli* CFUs/kg used material/m^2^) as a function of NPs loading in GWC IPs (in disks).

Figure 10 shows the *E. coli* growth in the presence of GWC IPs embedded with CuO and ZnO NPs after a 24 h incubation at 37 °C. It is evident that GWC IPs embedded with 0.5% and 1% CuO NPs partially eliminated *E. coli* growth. However, high bacterial populations were observed in samples of GWC IPs embedded with ZnO NPs. These results may be attributed to the low amount of 1% and 2% ZnO NPs that were used under the current experimental conditions.

Uncoated beads (GWC IPs, control) also had significant inhibition capacity (98%) for *E. coli* growth in comparison to *E. coli* growth solely in the liquid culture. IPs possess alkali pH values when in contact with aqueous media, suggesting inhibition of bacterial growth [66]. For that reason, acid washing of GWC IPs dishes was carried out; in general, high alkalinity of fresh concrete/mortar and/or IPs at an early age limit bacterial growth. However, the pH of IPs and/or concrete/mortar IPs may slowly be reduced over time by the effect of carbon dioxide and hydrogen sulfide gas and thus, bacteria may evolve [40]. Death of *E. coli* bacteria upon exposure to alkaline pH is expected. Exposure of Gram-negative bacteria such as *E. coli* to high pH (i.e., 0.025 M NaOH) destroys cell membranes and causes leakage of the internal contents of cells [67]. In addition, copper oxide nanorods prepared at 2.0 M NaOH concentration showed significant antibacterial potential against *Salmonella typhimurium* and *Staphylococcus aureus* strains [68]. Nano ZnO in metakaolin-based geopolymers may act as antibacterial agent limiting the growth of bacteria [45]. ZnO NPs coupled with a number of influenced factors such as particle size, concentration, morphology among others, confer significant toxicity to bacteria such as *E. coli* [69]. Flower-shaped (CuO NPs in this study) to rod-like and spherical (in this study) ZnO nanostructures exhibit higher antibacterial activity against *E. coli* [69]. However, in this study ZnO NPs did not exhibit antibacterial properties against *E. coli*. In agreement with a previous study [70] zinc oxide nanoparticles (of 21 nm) demonstrated no antibacterial activity against *E. coli.*

Prior to *E. coli* growth experiments, zinc ions in the leachates were not detected. These results are in line with Hg porosimetry measurements, in which ZnO NPs GWC IPs had higher bulk and/or apparent density and thus a more compact structure in comparison to CuO NPs GWC IPs. However, copper ions (1.1 and 1.8 mg∙L^−1^) in the leachates were determined for GWC IPs embedded with 0.5% and 1% CuO NPs, respectively. Release of ionic copper from copper-rich surfaces in the culture medium has also been suggested as a reason for their antimicrobial effect [71]. Leaching of copper ions from CuO NPs GWC IPs may explain the mechanism of action of CuO NPs. Metal ion release in relation to oxidative stress induction and/or non-oxidative mechanisms may occur simultaneously explaining NPs antibacterial behavior [72].

## 4. Conclusions

The effect of CuO- and ZnO-nanoparticles on the mechanical and structural properties of ground waste concrete waste was evaluated. GWC IPs embedded with 1% ZnO NPs showed a relative increase in compressive strength up to 23 MPa compared to GWC IPs with and without CuO NPs. The XRD patterns revealed that the addition of CuO and/or ZnO nanoparticles improved the interaction between NPs and inorganic polymer matrix, as indicated by shifts of the main peaks. Due to the poor content of aluminosilicate components in the raw material, limited inorganic polymerization is expected as was confirmed by Raman analysis. However, broadening of the main band can suggest the formation of differently structured material associated with the addition of NPs into IPs matrix. GWC IPs doped with 0.5% CuO NPs and 1% ZnO NPs exhibited a relatively dense structure and morphology with low water absorption. The homogeneity of a dense microstructure of NPs GWC IPs was evident from SEM imaging. The addition of NPs into GWC IPs matrix resulted in the augmentation of the mesoporosity of matrix thus a relative increase of compressive strength of the matrix was expected. Total intrusion volume of 1% ZnO NPs GWC IPs was lower due to the better void filling effect of ZnO NPs compared to CuO NPs. Porosity measurements were in agreement with compressive strength measurements. The incorporation of CuO and ZnO NPs suggested thermal stability and relatively improved thermal properties of IPs. GWC IPs embedded with 0.5% and 1% CuO NPs had positive antimicrobial properties that produced bacteria reduction as high as 80%.

Toward a circular economy, IPs binders are considered eco-friendly, non-hazardous, cost effective, and durable building materials that may have wide applications in repair and/or restoration cases in construction building and road materials and pavements. Development of carbonate material IPs have been less explored. Some benefits of using carbonate-rich wastes such as ground waste concrete in IPs consist of improving structural quality and load bearing and reducing maintenance cost and extending life. Besides the added benefit of waste management, the proposed IPs technology is an effective method for converting wastes into useful products. Additionally, IPs embedded with NPs may also be applied in functional surface applications such as floors and walls in houses and hospital facilities and/or marine concrete structures with antibacterial efficiency.

## Figures and Tables

**Table 1:** Chemical analysis of ground-waste concrete (GWC) (% weight).

Oxides/Material	SiO_2_	Al_2_O_3_	CaO	Na_2_O	Fe_2_O_3_	MgO	K_2_O	TiO_2_	MnO	P_2_O_5_	SO_3_	SrO	* LOI
GWC	3.96	0.99	54.43	1.80	0.51	1.95	0.07	0.05	0.01	0.04	0.46	0.13	35.05

* LOI stands for loss on ignition at 1050 °C for 4 h.

**Table 2:** Elemental composition for GWC IPs embedded with 0.5% CuO and 2% ZnO NPs.

Sample	GWC IPs		Cu0.5		Zn2	
Element	Weight %	Atomic %	Weight %	Atomic %	Weight %	Atomic %
Ca	26.91	13.60	22.75	10.92	15.85	7.39
Si	5.88	4.24	4.44	3.04	5.48	3.65
Na	3.36	2.97	2.62	2.19	4.05	3.30
Mg	1.30	1.08	1.33	1.05	1.59	1.23
Al	0.83	0.62	0.76	0.54	1.35	0.94
K	0.20	0.10	0.30	0.15	0.34	0.16
Cu	n.d.	n.d.	0.66	0.20	n.d.	n.d.
Zn	n.d.	n.d.	-	n.d.	0.03	0.01
O	61.12	77.39	68.12	81.91	71.35	83.32
Total	99.6	100	100.98	100	100.04	100

**Table 3:** Physical characteristics of GWC IPs synthesized with various amounts of CuO and/or ZnO replacements.

Sample No.	Specific Surface Area, m^2^·g^−1^	Total Pore Volume, TPV, mm^3^·g^−1^	Average Pore Diameter, APD, Å
Control	7.7	17	87.5
Cu0.5	6.5	20	124.6
Cu1	5.8	19	128.6
Cu2	8.1	26	129
Zn0.5	6.6	20	123
Zn1	8.0	22	108
Zn2	6.5	22	126

**Table 4:** Mercury intrusion porosimetry (MIP) analysis.

	Control	Cu0.5	Zn1
Total Pore Area, m^2^·g^−1^	4.946	5.676	4.844
Total Intrusion Volume, mL·g^−1^	0.155	0.158	0.112
Median Pore Diameter (Volume), µm	0.838	0.485	0.144
Median Pore Diameter (Area), µm	0.018	0.028	0.065
Average Pore Diameter (4V/A), µm	0.125	0.111	0.093
Bulk Density at 1.53 psia, g·mL^−1^	1.71	1.17	2.09
Apparent (skeletal) Density, g·mL^−1^	2.33	1.44	2.74
Porosity, %	26.5	18.5	23.5
Steam Volume Used, %	46	30	45

## Data Availability

Not applicable.
